# A Protocol for the International Translation and Validation of the Postpartum Specific Anxiety Scale for Preterm Birth [PSAS‐PTB] and Neonatal Intensive Care Unit [PSAS‐NICU] Contexts

**DOI:** 10.1002/mpr.70065

**Published:** 2026-02-13

**Authors:** Semra Worrall, Victoria Fallon, Paul Christiansen, Asma Khalil, Rosario Spencer, Andres Fresno, Francisca Wormald, Sandra Nakić Radoš, Ana Katušić, Mirna Kostović, Kateřina Azim Aburas, Suaad Moussa, Jaqueline Wendland, Giovanna M. Crivano, Ania Yahiaoui, Nikola Stenzel, Elana Payne, Anja Wittkowski, Mojgan Mirghafourvand, Sepideh Mashayekh‐Amiri, Tal Yatziv, Noa Gueron‐Sela, Alessandra Bramante, Chiara Ionio, Giulia Ciuffo, Gabija Jarašiūnaitė‐Fedosejeva, Erika Gibė, Viktorija Stankevičienė, Sana SaadAdeen, Helena Moreira, Brígida Caiado, Oana Luiza Rebega, Marta E. Aparicio‐García, Natalia Costas‐Ramón, Rosa M. Cárdaba‐García, Akindra Kariyawasam, Maheshika Maduwanthi, Ridma Thilakarathne, Sergio A. Silverio, Global Consortium for Perinatal Psychometrics

**Affiliations:** ^1^ Department of Psychology University of Liverpool Liverpool UK; ^2^ Department of Women & Children's Health King's College London London UK; ^3^ Fetal Medicine Unit St George's University Hospitals NHS Foundation Trust London UK; ^4^ Fetal Medicine Unit Liverpool Women's NHS Foundation Trust Liverpool UK; ^5^ Facultad de Psicologia Universidad de Talca Talca Chile; ^6^ Facultad de Medicina Pontificia Universidad Católica de Chile Santiago Chile; ^7^ Department of Psychology Catholic University of Croatia Zagreb Croatia; ^8^ Faculty of Education and Rehabilitation Sciences University of Zagreb Zagreb Croatia; ^9^ Department of Health Psychology University of Applied Health Sciences Zagreb Croatia; ^10^ Faculty of Arts Charles University Prague Czechia; ^11^ Department of Psychiatry Cairo University Cairo Egypt; ^12^ Laboratoire de Psychopathologie et Processus de Sante Université Paris Cité Paris France; ^13^ Psychologische Hochschule Berlin Berlin Germany; ^14^ Division of Psychology and Mental Health The University of Manchester Manchester UK; ^15^ Social Determinants of Health Research Center Tabriz University of Medical Sciences Tabriz Iran; ^16^ Department of Midwifery Golestan University of Medical Sciences Gorgan Iran; ^17^ Department of Psychology Ben‐Gurion University of the Negev Beersheba Israel; ^18^ Yale Child Study Center Yale University New Haven Connecticut USA; ^19^ Policentro Donna Milano Italy; ^20^ Dipartimento di Psicologia Università Cattolica del Sacro Cuore Milano Italy; ^21^ Department of Psychology Vytautas Magnus University Kaunas Lithuania; ^22^ Ministry of Health West Bank Palestine; ^23^ Faculty of Psychology and Education Sciences University of Coimbra Coimbra Portugal; ^24^ Department of Psychology Babeş‐Bolyai University Cluj‐Napoca Romania; ^25^ Department of Psychology Lucian Blaga University of Sibiu Subiu Romania; ^26^ Department of Social Work and Differential Psychology INSTIFEM Universidad Complutense de Madrid Madrid Spain; ^27^ Department of Physiatry and Nursing University of Zaragoza Zaragoza Spain; ^28^ Department of Nursing University of Valladolid Valladolid Spain; ^29^ Faculty of Nursing KIU University Colombo Sri Lanka

**Keywords:** cross‐cultural validity, NICU, postpartum anxiety, preterm birth, psychometrics, translation

## Abstract

**Objectives:**

To describe the process for the translation and validation of the Postpartum Specific Anxiety Scale—Preterm Birth [PSAS‐PTB] and the Postpartum Specific Anxiety Scale—Neonatal Intensive Care Unit [PSAS‐NICU].

**Methods:**

We outline a four‐stage process for translation and validation of the 10‐item PSAS‐PTB and the 16‐item PSAS‐NICU.

**Results:**

The protocol outlines a multi‐stage translation and validation process, including (1) independent forward translation, (2) independent back translation, (3) final approval following review, and (4) validity and reliability study.

**Conclusions:**

Culturally and contextually relevant assessment of postpartum anxiety is vital, particularly in vulnerable populations. Following standardised procedures will support broader applicability across diverse populations, providing a reliable self‐report tool for both research and clinical use.

## 1 Introduction

### 1.1 Postpartum Anxiety

Whilst some level of anxiety after birth is considered adaptive from a psychological perspective, for some women these anxieties can be debilitating and affect their daily life. Perinatal anxiety is characterised by excessive, severe, and irrational concerns (Wenzel 2014) during the perinatal period. Estimates suggest between 15% and 40% of women may experience postpartum anxiety (Dennis et al. 2017; Field 2018), with prolonged negative effects on both mother and infant, including infant feeding, bonding, and temperament (del Hoyo‐Bilbao and Orue 2024). As such, effective, accurate, and culturally appropriate measurement is vital to enable prevention strategies and early intervention (Harrison and Alderdice 2020; M. S. Smith et al. 2022), particularly given the intergenerational impacts of anxiety during this period (Walker et al. 2020).

To date, research has been limited by the use of general psychometric (self‐report or clinician administered) measures originally validated in general adult populations, such as the State Trait Anxiety Inventory (STAI; Spielberger 1983), the Generalised Anxiety Disorder Assessment—7‐item scale (GAD‐7; Spitzer et al. 2006), and the Edinburgh Postnatal Depression Scale anxiety subscale (EPDS 3‐A; Cox et al. 1987). Whilst these have been extrapolated for use in perinatal populations, often inappropriately (Alderdice 2020), they do not measure domains specific to the period after birth, such as feeding, sleep, and infant routine, which are well established as significant sources of anxiety (Astbury et al. 2025; Sun et al. 2020). Therefore, this lack of domain‐specific measurement may lead to an underestimation of anxiety in this specific population and/or for perinatal anxiety to be diagnostically overshadowed by perinatal depression (Harris et al. 2024).

In response to the lack of appropriate measures specifically for use in this population, the Postpartum Specific Anxiety Scale (PSAS; Fallon et al. 2016) was developed. This self‐report scale consists of 51 items across four factors rated on a Likert scale from 1–4, with these subscales measuring anxieties relating to maternal competence and attachment, infant safety and welfare, practical infant care, and psychosocial adjustment to motherhood. Higher scores indicate higher levels of anxiety. Subsequently, a 16‐item Research Short Form (PSAS‐RSF; Davies et al. 2021) and a 12‐item Research Short Form for use during crises (PSAS‐RSF‐C; Silverio et al. 2021) were developed and validated for use in English‐speaking mothers. All of the English language derivatives of the PSAS have excellent validity and reliability and are better predictors of maternal and infant health outcomes compared to generalised measures (Davies et al. 2022; Fallon et al. 2018, 2022).

To date, the measure, and its derivatives, have been officially translated and validated in French (PSAS‐FR; Infante‐Gil et al. 2022), Italian (PSAS‐IT; Ionio et al. 2023), Spanish (PSAS‐ES; Costas‐Ramón et al. 2023), Brazilian Portuguese (PSAS‐BR; Souza Torres de Araújo et al. 2024), Persian (PSAS‐IR; Hasanzadeh et al. 2021; PSAS‐IR‐RSF; Mashayekh‐Amiri, Jafarabadi, Davies, et al. 2023; PSAS‐IR‐RSF‐C, Mashayekh‐Amiri, Jafarabadi, Montazeri, et al. 2023), Mandarin Chinese (PSAS‐CN; Xu et al. 2021), and Jordanian Arabic (PSAS‐JO‐RSF; Hijazi et al. 2024). Ongoing translations are currently being undertaken in Germany, The Netherlands, Portugal, Palestine, Greece, Slovakia, Croatia, Sweden, Myanmar, India, Egypt, Czechia, Türkiye, Indonesia, Sri Lanka, Lithuania, Israel, and Chile; as well as other derivative translations in the countries which already have at least one published version.

### 1.2 Maternal Anxiety in High‐Risk Populations

Mothers who have given birth prematurely (< 37 weeks' gestation) and/or who have had an infant in the Neonatal Intensive Care Unit [NICU] are particularly susceptible to mental health difficulties in the postpartum period, with childbirth‐related posttraumatic stress disorder (PTSD) being four times more prevalent in high‐risk samples than in the community samples of mothers (Dikmen Yildiz et al. 2017). The most recent estimates suggest the global prevalence of preterm birth is approximately 9.9% (Ohuma et al. 2023). There is wide disparity in rates of preterm birth, both between‐ and within‐countries (Delnord et al. 2015; L. Smith et al. 2025), with rates highest in Southern Asia and sub‐Saharan Africa (Ohuma et al. 2023).

Despite concerted efforts to identify the causes of preterm birth and to outline avenues for prevention (Care et al. 2022; Khandre et al. 2022), in all regions globally preterm birth rates have plateaued and remained unchanged throughout the past decade (Bradley et al. 2025). As such, the psychological burden of preterm birth remains substantial, and attention must also be directed towards the psychological impacts, given there has been no measurable decrease in rates of mental health difficulties, particularly in anxiety symptoms that are known to be more prevalent in mothers who give birth prematurely (Worrall et al. 2023). These anxieties may be further exacerbated in women who have infants admitted to the NICU (Galea et al. 2022), in part due to the increased health concerns associated with these infants, early parent‐infant separation, and increased financial constraints with a prolonged hospital admission (Lakshmanan et al. 2022; Trumello et al. 2018).

Although some other measures have been developed for parents of preterm babies, such as the Preterm Birth Experience and Satisfaction Scale (B‐BESS; Sawyer et al. 2014), they are not focused specifically on postpartum anxiety. Whilst the PSAS (Fallon et al. 2016) and the PSAS‐RSF (Davies et al. 2021) have been used in two studies exploring the relationship between anxiety and gestational age at birth (Worrall et al. 2023, 2024a), mothers of premature infants have been found to respond differently to items on the PSAS‐RSF than mothers of term infants (Worrall et al. 2024b), and none of the items were found to capture the distinct circumstances experienced by women with premature infants or those whose babies are admitted to the NICU (Worrall et al. 2025). In response, the Postpartum Specific Anxiety Scale—Preterm Birth [PSAS‐PTB] and the Postpartum Specific Anxiety Scale—Neonatal Intensive Care Unit [PSAS‐NICU] have recently been developed (Worrall et al. 2025), with 10 and 16 items, respectively. Despite the broad global interest in the PSAS to date, no formal, standardised procedure for the translation and subsequent validation has yet been written. This protocol is designed to address this deficit, which currently limits global comparability and use in clinical and research settings. This is of particular importance within the context of preterm birth and NICU admission, where the likelihood of developing a perinatal mental health disorder is higher. It has also been recently recommended that when translations of measures are to be used, this process should be made clear in a protocol to ensure transparency (Ozolins et al. 2020).

### 1.3 Aims

In this protocol, we aim to outline the procedure for the translation and subsequent validation of the PSAS‐PTB (10‐items) and the PSAS‐NICU (16‐items). Specifically, we will outline the procedures to (a) translate the PSAS‐PTB and PSAS‐NICU into the target language (depending upon the country) using standardised translation and back‐translation procedures and adapt both scales for the target population (i.e., mothers of premature and/or mothers of infants in the NICU) to ensure cultural and linguistic sensitivity and suitability and (b) assess the psychometric properties (e.g., reliability, validity) of the translated versions.

## 2 Methods

### 2.1 Overarching Study Design

We are adopting a pragmatic philosophical approach to both ontology and epistemology due to the positivist nature of psychometric scale outcomes, but with the acceptance that culture and country will have a bearing on individual items' interpretation. The translation and validation of the scales are discussed separately because they comprise of different methodologies: first the translation of the scales (Stage 1: Independent Forward Translation, Stage 2: Independent Back Translation and Stage 3: Final Approval) and validation of the translated versions (Stage 4: Validation Study). The procedure follows an established process, which has been outlined to translate other versions of the PSAS globally, which is also consistent with a recent review outlining translation and subsequent validation of scales into other different languages (Cruchinho et al. 2024) and World Health Organization (WHO) guidance on translation of health measures (WHO, n.d.). This is also in line with COSMIN guidelines on translation patient‐reported outcome measures (Mokkink et al. 2019).

## 3 Proposed Procedure

As has always been the case with all derivatives of the PSAS, the English‐language versions of the tool should be published first before any versions in other languages, to ensure that the translated items are, in fact, translations of the final validated items of the PSAS‐PTB and the PSAS‐NICU. For the purposes of this protocol, the following languages have been agreed: Chilean Spanish [RS; AF; FW], Croatian [SNR; AKat; MK], Czech [KAA], Egyptian Arabic [SM], French [JW; GMC, AY], German [NS; EP; AW], Persian [MM; SM‐A], Hebrew [TY; NG‐S], Italian [AB; CI; GC], Lithuanian [GJ‐F; EG; VS], Palestinian Arabic [SSA], Portuguese [HM; BC], Romanian [OLR], Spanish [MEA‐G; NCR; RMC‐G], and Sinhala [AKar; MMa; RT]. Other countries will be permitted to apply to translate and validate into their own languages hereinafter. These translations and validations will be conducted in conjunction with the Management Committee of the Global Consortium for Perinatal Psychometrics. At the time of writing, this consists of a psychometrician [PC], a perinatal psychologist [VF], a social and implementation scientist [SAS], and a doctoral student who oversees the day‐to‐day management of the Consortium [SW]. The Management Committee will be open to new members from across the Consortium as the Consortium grows.

Should individual study teams choose to do so, they can validate only one of the scales. However, given how closely related the two populations of interest are, and to reduce burden on each individual study team, it is envisioned that both scales will be translated simultaneously. This is because most preterm infants will spend some period of time in NICU care, and therefore both tools will likely be required, hence the simultaneous translation and validation is advised. For a simplified version of the process, see Table 1.

**TABLE 1:** Overview of translation and validation stages for the PSAS‐PTB and PSAS‐NICU.

Stage	Activity	Responsible team	Key outputs/Deliverables
Stage 1: Independent forward translation	Three independent bilingual translators (fluent in English and the target language, with expertise in perinatal mental health, preterm birth, or NICU) translate the PSAS‐PTB and/or PSAS‐NICU into the target language.	Local country study team	Three forward‐translated versions of each scale. Notes on cultural or linguistic issues.
Stage 2: Independent back translation	A fourth independent bilingual expert, blinded to the original scales, back‐translates the selected target‐language versions into English.	Independent back‐translator	Back‐translated English versions of the scales.
Stage 3: Harmonisation and final approval	Comparison of the back‐translated and original English versions by the local study team and the global consortium for perinatal psychometrics. Revisions made to ensure conceptual and linguistic equivalence.	Local team & the Management Committee of the Global Consortium for Perinatal Psychometrics	Final approved target‐language versions of the PSAS‐PTB and/or PSAS‐NICU.
Stage 4: Cultural adaptation (optional)	If required, conduct focus groups, expert panels, or cognitive interviews to adapt or refine items for cultural relevance.	Local study team; expert stakeholders in collaboration with the Management Committee of the Global Consortium for Perinatal Psychometrics	Refined culturally appropriate versions; documentation of any item modifications.
Stage 5: Validation study	Administration of the final translated scales to eligible participants (mothers of preterm or NICU infants). Psychometric analyses conducted.	Local country study team	Validated target‐language PSAS‐PTB and/or PSAS‐NICU scales. Psychometric dataset and report.
Stage 6: Cross‐cultural measurement invariance (international phase)	Aggregated international data used to examine measurement invariance across countries.	Management Committee of the Global Consortium for Perinatal Psychometrics	Evidence of cross‐cultural comparability and international validation of PSAS‐PTB and PSAS‐NICU.

### 3.1 Independent Forward Translation (English → Target Language)

Forward translation is the initial step in the translation process (Yu et al. 2004), which should be undertaken by three independent translators who are fluent in both English and the target language and ideally have knowledge of perinatal mental health and/or the preterm birth/NICU contexts and research areas. Where possible, the translation team should be comprised of more than one disciplinary background (i.e., psychology, medicine, midwifery, etc.) to ensure both technical accuracy and cultural appropriateness: and should have a clinical and/or academic background, preferably with at least one of the translators being a senior researcher. Each study team will be responsible for finding the independent translator. Each translator should work independently to produce a version of the PSAS‐PTB and/or the PSAS‐NICU, in the target language. At this stage, if any of the independent translators believe items need to be adapted, removed, or added, they can do so in collaboration with the Management Committee of the Global Consortium for Perinatal Psychometrics.

### 3.2 Independent Back Translation (Target Language → English)

Once three independent versions of the PSAS‐PTB and/or the PSAS‐NICU have been completed, these are handed to a fourth, independent member of the team blind to the original scale to complete the back translation. They should, again, be fluent in English and the target language, but not necessarily familiar with the scale or its purpose, yet still have expertise aligned to perinatal mental health, preterm birth, or NICU admissions. The role of the back‐translator is to assess the three translations and select which version of each item on the scale/s is the most appropriate for the target language. The person back‐translating will be responsible for making decisions about which of the three versions of every item will be selected, but also will be responsible for harmonising language across all items and therefore will make decisions not only based on eloquence, but also consistency (see Ozolins et al. 2020).

### 3.3 Final Approval

Once translated back into English, the back‐translated version of the PSAS‐PTB and/or the PSAS‐NICU should now be compared to the original English‐language PSAS‐PTB and PSAS‐NICU, respectively. This procedure will be undertaken by a larger team, including the local study team, original translators, and the Management Committee of the Global Consortium for Perinatal Psychometrics. Discrepancies or inconsistencies between the back translation and the original scale(s) should be discussed to facilitate resolution and harmonisation. These discrepancies may be in concepts, such as ambiguous wording or lack of specificity, and forward translation can be refined and augmented to address any of these potential issues. Once agreed, this version will be the final translated version of the PSAS‐PTB and the PSAS‐NICU in the target language.

### 3.4 Adaptation

If at this stage, it is identified that items will differ in different cultural contexts, or that they may need adapting, or removing completely, the respective country team may wish to undertake focus groups with stakeholders, expert panel review, or cognitive interviewing with the translated version. These steps are outlined in a previously published protocol describing the original development of the scales (Worrall et al. 2025). Any modification, removal, or addition of items to ensure cultural applicability should be undertaken in collaboration with the Management Committee of the Global Consortium for Perinatal Psychometrics.

### 3.5 Validation Study

The final step in the development of any psychometric scale is to ensure comprehensive validity and reliability testing (e.g., convergent and divergent validity, test‐retest reliability). This has traditionally been conducted on‐line, but it can also be collected face‐to‐face (e.g., Fallon et al. 2016; Davies et al. 2021). The study team should aim to validate the scale(s) in mothers of infants up to 12 months corrected age, but if local resource does not allow for this, we would recommend at least validating up to 6 months corrected age; as has been the case with other derivatives of the PSAS. For example, the original PSAS was initially validated in mothers of infants aged up to 6 months (Fallon et al. 2016) and was later validated in mothers of infants aged up to 12 months (Fallon et al. 2022).

Individual study teams will be responsible for gaining their own ethical approval, and for collecting the data. Each national research team will store data securely on institutional servers compliant with General Data Protection Regulation (GDPR) and local data protection laws. De‐identified datasets will be submitted to a central repository managed by the Global Consortium for Perinatal Psychometrics. Participants will receive full study information in their native language and provide informed consent electronically or in writing. Given the potentially sensitive nature of the questions asked, participants who indicate high levels of anxiety or distress will be provided with referrals to local psychological or support services in accordance with national protocols. Eligible participants will be required to be over the age of 18, with a good understanding of the target language, who either have a premature infant (< 37 weeks' gestation) now aged between 0–6/12 months corrected age (chronological age minus gestational age), who has/has not previously spent time in the NICU (as recognised by the country), or a live infant born at term now aged between 0 and 12 months, who is/or has previously spent time in the NICU. Screening questions can be used if the study is administered online to ensure participants meet eligibility criteria. Previous PSAS validations have traditionally recruited these participants on‐line via social media. However, given the clinical nature of the scale(s), individual study teams may wish to recruit using different modalities.

Depending upon resource in each given country, teams may choose to conduct a pilot test on the survey. This can be done with expert panel groups, and cognitive interviews as has previously been done with the PSAS (see Worrall et al. 2025) and is not subject to a pre‐full validation pilot study test.

Study teams should aim to recruit approximately 200 participants for a Confirmatory Factor Analysis [CFA] as a minimum (Bryant and Yarnold 1995; Hair and et al. 1998), because we expect there to be high factor loadings and high communalities between items. These sample sizes will also be adequate for the assessment of internal reliability of the scales (i.e., McDonald's Omega).

It is recommended that participants will complete demographic questions as appropriate (e.g., medical diagnoses, length of stay in the NICU, medical complications, mode of birth etc.) and approved by each countries' ethics committee, the PSAS‐RSF (Davies et al. 2021), the PSAS‐PTB (if applicable), the PSAS‐NICU (if applicable). The UK‐based validation study is utilising a battery of other psychometric measures including the GAD‐7 (Spitzer et al. 2006), EPDS (Cox et al. 1987), the Postpartum Bonding Scale (PBQ; Brockington et al. 2001), the last 10 items of the Revised Infant Temperament Questionnaire (RITQ; Carey and McDevitt 1978), the Perceived Maternal Parenting Self‐Efficacy Scale (PMPSES; Barnes and Adamson‐Macedo 2007Barnes and Adamson‐Macedo 2007), and the Parental Stressor Scale: Neonatal Intensive Care Unit (PSS:NICU; Miles et al. 1993; if applicable). Individual country teams may wish to include these measures in the target language if available. It is also recommended the study teams employ a follow‐up survey 2 weeks later to establish test‐retest reliability, which consists of the PSAS‐RSF, the PSAS‐PTB, the PSAS‐NICU (if applicable), at minimum, alongside the anxiety and depression measures used in the original survey. Study teams may also choose to add additional measures as outcomes in their target language as they see fit.

As response options on the PSAS are ordinal, the EFA should be conducted on the polychoric correlation matrix whilst the CFA should use either a diagonally weighted least squares estimator or maximum likelihood with robust standard errors. McDonald's Omega (total for subscales; and hierarchical for total scores) will be used to assess internal reliability. Convergent and divergent validity with other outcome measures will be tested using correlations. Should individual study teams face difficulty with recruitment, there is the possibility to conduct a pilot study on the measures (minimum *n* = 20), however should be cognisant that this does not replace a full validation of the measures.

Once data from several countries has been obtained and collated in an open data repository, there is scope to conduct a measurement invariance analysis across countries, which has previously been done using the PSAS in mothers of premature infants (Worrall et al. 2024b), and has been recommended by COSMIN (Mokkink et al. 2019). In short, this consists of testing whether interpretations of the scale can be meaningfully compared across groups (in this case, countries). This includes testing configural invariance (to ensure that the factor structure is consistent), metric invariance (to assess whether each individual item on the scale performs in the same way across countries), and scalar invariance (to compare means of factor loadings), and finally strict invariance (to determine if the items' unique variances are consistent across groups). This is an iterative process, meaning that each stage of the analysis is only performed if the assumptions of the previous stage are met.

## 4 Discussion

In this protocol, we outline the process for the translation and subsequent psychometric validation of the PSAS‐PTB and the PSAS‐NICU. By appropriately and accurately translating these measures, we aim to provide clinicians and researchers with a tool to assess anxiety in the unique contexts of premature birth and the NICU environment. To our knowledge, the PSAS‐PTB and the PSAS‐NICU are the first postpartum‐specific measures of anxiety developed for these populations. The methodological approach for translation and validation follows established protocols (Mokkink et al. 2019), which will further enhance their accuracy, relevance, and acceptability. Developing culturally and contextually relevant tools is critical for improving both screening and intervention programmes, which will improve outcomes for both mothers and their infants because the use of a domain‐specific tool can allow for targeted intervention. It should also be noted that concerted efforts must be made to recruit participants from diverse and minoritised backgrounds including those with socially complex lives, ethnic and sexual minorities, migrant populations, those with disabilities, and those who find healthcare hard to access, as well as LGBTQ+ families, and others with diverse family structures.

Whilst we outline a rigorous process for translation, study teams should be cognisant that translation may present challenges, such as ensuring semantic equivalence, verifying meaning of items is not lost, and that items may need to be adapted for cultural relevance. As such, this protocol further highlights the importance of following an established process to ensure valid, standardised, linguistic and psychometric validity.

## Author Contributions

**Semra Worrall:** conceptualisation, methodology, visualisation, writing – original draft. **Victoria Fallon:** methodology, supervision, writing – review and editing. **Paul Christiansen:** methodology, supervision, writing – review and editing. **Asma Khalil:** supervision, writing – review and editing. **Rosario Spencer:** writing – review and editing. **Andres Fresno:** writing – review and editing. **Francisca Wormald:** writing – review and editing. **Sandra Nakić Radoš:** writing – review and editing. **Ana Katušić:** writing – review and editing. **Kateřina Azim Aburas:** writing – review and editing. **Suaad Moussa:** writing – review and editing. **Jaqueline Wendland:** writing – review and editing. **Giovanna M. Crivano:** writing – review and editing. **Ania Yahiaoui:** writing – review and editing. **Nikola Stenzel:** writing – review and editing. **Elana Payne:** writing – review and editing. **Anja Wittkowski:** writing – review and editing. **Mojgan Mirghafourvand:** writing – review and editing. **Sepideh Mashayekh‐Amiri:** writing – review and editing. **Tal Yatziv:** writing – review and editing. **Noa Gueron‐Sela:** writing – review and editing. **Alessandra Bramante:** writing – review and editing. **Chiara Ionio:** writing – review and editing. **Giulia Ciuffo:** writing – review and editing. **Gabija Jarašiūnaitė‐Fedosejeva:** writing – review and editing. **Erika Gibė:** writing – review and editing. **Viktorija Stankevičiene:** writing – review and editing. **Sana SaadAdeen:** writing – review and editing. **Helena Moreira:** writing – review and editing. **Brígida Caiado:** writing – review and editing. **Oana Luiza Rebega:** writing – review and editing. **Marta E. Aparicio‐García:** writing – review and editing. **Natalia Costas Ramón:** writing – review and editing. **Rosa M: Cárdaba‐García:** writing – review and editing. **Akindra Kariyawasam:** writing – review and editing. **Maheshika Maduwanthi:** writing – review and editing. **Ridma Thilakarathne:** writing – review and editing. **Sergio A. Silverio:** conceptualisation, methodology, project administration, writing – review and editing.

## Funding

Miss Semra Worrall's PhD is supported financially by the University of Liverpool; however, the institution has no influence over research design, data collection, analysis, write‐up or the decision to submit. All other authors declare no known competing financial interests or personal relationships which could have appeared to influence the work reported in this article.

## Ethics Statement

The authors have nothing to report.

## Conflicts of Interest

The authors declare no conflicts of interest.

## Data Availability

The authors have nothing to report.
